# Hereditary Transthyretin Amyloidosis: Genetic Characterization of the *TTR* P.Val142Ile Variant in a Calabrian Kindred

**DOI:** 10.3390/genes16080960

**Published:** 2025-08-14

**Authors:** Francesca Dinatolo, Radha Procopio, Valentina Rocca, Elisa Lo Feudo, Adele Dattola, Lucia D’Antona, Fernanda Fabiani, Emma Colao, Rosario Amato, Francesco Trapasso, Giuseppe Viglietto, Rodolfo Iuliano

**Affiliations:** 1 Medical Genetics Unit, Renato Dulbecco University Hospital, 88100 Catanzaro, Italy; francesca.dinatolo@studenti.unicz.it (F.D.); valentina.rocca@unicz.it (V.R.); elisa.lofeudo@studenti.unicz.it (E.L.F.); adeledatto@gmail.com (A.D.); dantona@unicz.it (L.D.); f.fabiani@materdominiaou.it (F.F.); colaoemma@unicz.it (E.C.); rosario.amato@unicz.it (R.A.); trapasso@unicz.it (F.T.); viglietto@unicz.it (G.V.); iuliano@unicz.it (R.I.); 2 Department of Medical and Surgical Sciences, Neuroscience Research Center, Magna Graecia University, 88100 Catanzaro, Italy; 3 Department of Experimental and Clinical Medicine, Campus S. Venuta, University Magna Graecia of Catanzaro, 88100 Catanzaro, Italy; 4 Department of Health Sciences, Campus S. Venuta, University Magna Graecia of Catanzaro, 88100 Catanzaro, Italy

**Keywords:** hereditary transthyretin amyloidosis, *TTR* gene, p.Val142Ile

## Abstract

Background: Hereditary transthyretin amyloidosis (ATTRv) is a systemic disorder caused by homozygosity or compound heterozygosity for pathogenic mutations in the *TTR* gene, leading to destabilization of the transthyretin tetramer, misfolding of monomers, and subsequent amyloid fibril deposition. Among over 150 known *TTR* variants, p.Val142Ile is particularly associated with late-onset cardiac involvement and is the most prevalent amyloidogenic mutation in individuals of African and, to a lesser extent, European descent. This study reports the identification and familial segregation of the p.Val142Ile mutation in a large multigenerational family from Calabria (Southern Italy). Methods: Genomic DNA was extracted from peripheral blood, and Sanger sequencing of the *TTR* gene was performed in the proband and extended family. Results: The proband was a 75-year-old man with clinical features suggestive of cardiac amyloidosis. Genetic testing revealed homozygosity for the *TTR* p.Val142Ile variant. Family screening revealed multiple heterozygous carriers across three generations, most of whom were asymptomatic. Discussion: This is the first report of a native Calabrian family carrying this variant, previously unreported in this region, where p.Phe84Leu was considered the only endemic *TTR* mutation. Our findings expand the mutational landscape of ATTRv in Southern Italy and highlight the presence of p.Val142Ile in a previously unrecognized geographic area. These results reinforce the importance of including *TTR* sequencing in the work-up of unexplained cardiomyopathy, particularly in Southern Italy, where atypical variants may be emerging.

## 1. Introduction

Amyloidosis encompasses a group of rare and heterogeneous disorders defined by the extracellular deposition of insoluble and misfolding proteins, known as amyloid fibrils [1]. These fibrils are formed through globular and soluble proteins, which undergo structural misfolding into β-pleated sheet conformations, rendering them hydrophobic, insoluble, non-functional, and resistant to proteolytic degradation [2,3]. The progressive accumulation of these misfolded proteins disrupts tissue architecture and impairs organ function, frequently affecting organs such as the heart, kidneys, and skin. In particular, cardiac involvement is a hallmark of many forms of amyloidosis and should be suspected in patients presenting with heart failure symptoms characterized by signs of right ventricular dysfunction, including lower extremity edema, jugular vein distension, hepatic congestion, ascites, and dyspnea [4]. Furthermore, conduction system involvement by amyloid deposits can lead to syncope or pre-syncope, complicating the clinical presentation.

The process of amyloid fibril formation, or fibrillogenesis, may result from sustained overproduction of proteins, intrinsic instability of wild-type proteins, or proteolytic cleavage yielding amyloidogenic fragments. Under normal conditions, cellular and extracellular quality control systems manage protein folding and eliminate misfolded species. However, when these mechanisms are compromised—due to genetic predisposition, environmental factors, or age-related decline—protein aggregation ensues. This leads to the formation of intermediate protofibrils and eventually mature fibrils, which are associated with proteotoxic effects even at early stages of aggregation [2,3]. Despite significant advances in diagnostic tools and a growing understanding of protein misfolding, the precise molecular events that trigger and sustain amyloid formation remain incompletely understood.

More than 40 amyloidogenic proteins have been identified to date, each capable of forming pathogenic deposits either locally or systemically [5,6].

Among them, transthyretin, encoded by the *TTR* gene, is a central amyloidogenic protein involved in two distinct forms of systemic amyloidosis: wild-type transthyretin amyloidosis (ATTRwt), previously known as senile systemic amyloidosis, and hereditary transthyretin amyloidosis (ATTRv), formerly referred to as familial amyloid polyneuropathy [7].

ATTRwt amyloidosis is a sporadic form of the disease resulting from the age-related misfolding and aggregation of structurally normal transthyretin. It predominantly affects elderly individuals and is often associated with cardiac involvement, although phenotypic overlaps with hereditary forms can occur [8].

Conversely, ATTRv amyloidosis is caused by pathogenic variants in the *TTR* gene and represents the most common form of hereditary systemic amyloidosis. It follows an autosomal dominant inheritance pattern and typically arises from a single amino acid substitution that destabilizes the native tetrameric structure, promoting amyloid fibril formation [9]. The clinical phenotype is highly variable and may include progressive sensorimotor polyneuropathy, autonomic dysfunction, cardiomyopathy, and gastrointestinal symptoms. If left untreated, the disease typically progresses rapidly, leading to death within approximately 10 years of symptom onset.

Globally, the prevalence of ATTRv is estimated at approximately 50,000 individuals, although clinical expression and severity vary widely among affected populations [10].

Transthyretin is predominantly synthesized by hepatocytes, with additional expression in the choroid plexus of the brain, retinal pigment epithelium, and pancreatic islet α-cells [11]. Functionally, transthyretin acts as one of three major transporters of thyroxine (T4), alongside albumin and thyroxine-binding globulin, and one of the transporters of retinol (vitamin A), through binding to retinol-binding protein (RBP) [12].

Transthyretin is encoded by the *TTR* gene as a 147-amino acid polypeptide, of which the first 20 residues constitute a signal peptide that is cleaved during processing prior to secretion. The mature protein consists of 127 amino acids and assembles into a highly conserved homotetramer (with a central channel). Each subunit comprises eight antiparallel β-strands (A-H), forming two β-sheets (DAGH and CBEF). Tetramer stability is maintained by hydrogen bonds between monomers and by hydrophobic interactions between dimers. Despite the presence of two T4 binding sites on the tetramer, only one molecule is typically bound due to negative cooperativity. Similarly, although the tetramer has four binding sites for RBP, it binds only two RBP molecules [13].

**Figure 1:**
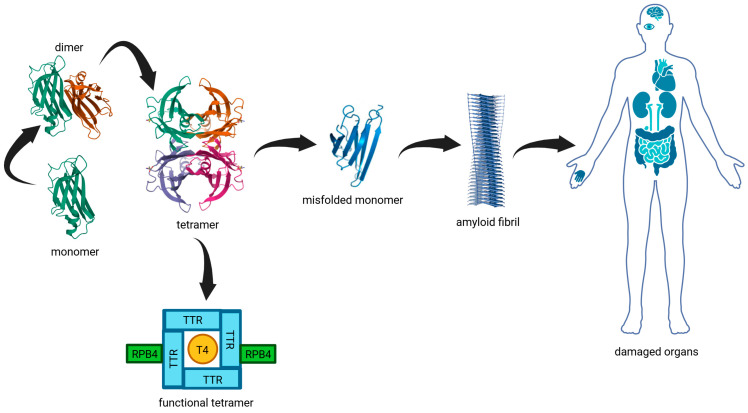
Graphical summary of TTR structure, stabilization, misfolding pathway, and organ involvement in ATTRv. Native TTR circulates as a functional tetramer composed of four identical subunits, responsible for transporting T4 and RBP4. Destabilization of the tetramer leads to dissociation into monomers, which can misfold and aggregate into amyloid fibrils. These fibrils deposit in various organs—most commonly the heart, peripheral nerves, kidneys, gastrointestinal tract, and central nervous system—causing progressive dysfunction. This figure was created with BioRender and incorporates crystallographic data of human TTR (PDB ID: 4TLT).

A graphical summary of the TTR structure, its physiological function, the pathogenic misfolding cascade, and the main organs involved in ATTRv is provided in Figure 1.

Pathogenic mutations in the *TTR* gene result in reduced stability of the native tetrameric structure of transthyretin, leading to the dissociation of the tetramer [14]. These monomers can subsequently misfold and aggregate into insoluble amyloid fibrils.

To date, more than 150 distinct mutations have been identified in the *TTR* gene [15]. The p.Val50Met was the first mutation linked to ATTRv, initially reported in endemic regions of Portugal, Japan, and Sweden [16] and then worldwide [17]. The Val50Met variant is described in the literature as Val30Met. Val50Met is referred to as the position in the complete sequence of the protein (including 20 aa of the signal peptide). The Val30Met variant refers to the substitution of a Valine with a Metionine at the 30 position of the TTR mature protein. Notably, its phenotypic expression is influenced by geographic origin: in Portugal, Brazil, and some Japanese populations the mutation is typically associated with early-onset disease (20–40 years) and high penetrance. In contrast, patients from Sweden and non-endemic areas in Japan often exhibit late-onset disease (>50 years) with reduced penetrance. The underlying causes of these disparities remain poorly understood, though founder effects and genetic modifiers have been proposed.

Another clinically relevant variant, p.Val142Ile (classically p.Val122Ile), is found in approximately 3% of African American individuals and 5% of the West African population. This mutation is strongly associated with late-onset ATTRv amyloid cardiomyopathy and represents the second most prevalent pathogenic *TTR* variant among individuals from a series of 23 countries worldwide, excluding the whole African continent and other large countries such as China, Russian, and India [18].

Table 1 summarizes the geographic distribution of the most frequently reported pathogenic TTR variants associated with ATTRv.

Interestingly, certain mutations like Arg104His and Thr119Met may confer a protective effect by enhancing tetramer stability. Compound heterozygotes (e.g., p.Val50Met/p.Thr139Met or p.Val50Met/p.Arg124His) typically exhibit a slower disease progression with milder symptoms compared to p.Val50Met homozygotes [20].

Here, we present the results of a comprehensive genetic analysis performed in a large multigenerational family from Calabria (Southern Italy), contributing to the characterization of the molecular basis of ATTRv in this population.

## 2. Materials and Methods

The study was approved by the “Comitato Etico Territoriale Regione Calabria” (Protocol n. 49/2025 of 27 February 2025) and was conducted in accordance with the Declaration of Helsinki and institutional ethical standards. Patients were recruited at the Medical Genetics Unit of the Renato Dulbecco University Hospital in Catanzaro, Italy. Written informed consent for genetic testing and data usage was obtained from all participants.

Clinical, cardiologic, and laboratory evaluations were performed as part of routine diagnostic procedures.

Peripheral blood samples were collected from the proband and consenting relatives. Genomic DNA was extracted using a commercial kit (QIAamp DNA Blood Mini Kit, Qiagen, Hilden, Germany), following the manufacturer’s instructions. Genetic analysis of the *TTR* gene was performed by Sanger sequencing. All four coding exons and flanking intronic regions were amplified by polymerase chain reaction (PCR) using specific primers. Sequencing was carried out using the SeqStudio Genetic Analyzer (Applied Biosystems, Thermo Fisher Scientific, Waltham, MA, USA). Sequence data were compared to the *TTR* reference sequence NM_000371.4 using standard bioinformatics tools. Upon identification of a pathogenic *TTR* variant in the proband, cascade genetic testing was offered to first- and second-degree relatives. Molecular analysis focused on targeted screening for the c.424G>A (p.Val142Ile) variant. Clinical information and relevant comorbidities were collected from family members who consented to testing.

Illustrative figures were created using BioRender (https://www.biorender.com/).

## 3. Results

The proband was a 75-year-old male who presented for a cardiological consultation with bilateral carotid atherosclerosis and a diagnosis of atrial flutter (AF), for which he was undergoing treatment with oral anticoagulants. Additionally, he had type 2 diabetes treated with oral hypoglycemics. The blood tests were within normal limits, except for glucose at 111 mg/dL, troponin T hs at 29.0 ng/L, and NT-proBNP at 1173 pg/mL. The electrocardiogram (ECG) showed a sinus rhythm, with normal atrioventricular conduction interval, ventricular ectopic beats, left anterior hemiblock, and diffuse abnormalities in ventricular repolarization.

The echocardiographic examination revealed a normal diameter of the ascending aorta, with thickened walls of the aortic root and left ventricle, and an enlargement of the left atrium. There was an increase in the wall thickness with concentric hypertrophy of the left ventricle, and the interventricular septum exhibited a “granular sparkling” appearance. Hypokinesia was noted in the middle-basal anterior wall, with left ventricular systolic function indices at the lower limits of normal. Overall, there were alterations in the fragmentary kinetics with low left ventricular systolic function indices and moderate mitral–tricuspid insufficiency.

The resulting picture led the specialist to a diagnosis of non-obstructive hypertrophic cardiomyopathy, with a suspected underlying diagnosis of cardiac amyloidosis (ATTR). The patient was subsequently referred to our department for genetic evaluation to investigate the potential presence of pathogenic variants related to ATTR.

Sanger sequencing of the *TTR* gene revealed that the proband, whose parents were not known to be consanguineous and whose family was not suspected to have any African origins, was homozygous for the c.424G>A (p.Val142Ile) variant. Unfortunately, the proband subsequently died; however, no further details regarding the circumstances or cause of death could be obtained, as he had been managed by the Cardiology Department, which had provided the blood sample for genetic analysis, and medicolegal restrictions prevented access to additional clinical information.

Targeted genetic testing was then extended to the proband’s family. The initial request for targeted analysis of the known variant was made by four out of the proband’s five siblings.

One sister, affected by Hashimoto’s thyroiditis and hypercholesterolemia, was found to be heterozygous for the familial variant. Another sister, affected by hypercholesterolemia, tested negative. A third sister, with a history of hypotension, was found to be heterozygous for the familial variant. One brother, in apparently good health, also tested heterozygous for the familial variant. The remaining brother, evaluated at a later time, presented a clinical history suggestive of arterial hypertension, hypercholesterolemia, cerebral ischemia, type 2 diabetes mellitus, and recently detected elevated microalbuminuria levels; he was likewise found to be heterozygous for the familial variant.

Subsequently, the proband’s four children, all aged between 40 and 50 years at the time of the investigation, underwent genetic testing. All were confirmed to be obligate heterozygotes, consistent with Mendelian inheritance, and this was corroborated by molecular analysis. At the time of clinical evaluation, none of them exhibited cardiac symptoms, with the exception of one daughter who reported recurrent extrasystoles.

Segregation analysis was subsequently extended to the children of the proband’s siblings who were found to be heterozygous, leading to the identification of two additional heterozygous individuals.

Furthermore, four of the proband’s cousins (two offspring of the proband’s paternal aunt and two offspring of the proband’s paternal uncle) voluntarily presented for genetic counseling, resulting in the identification of one more heterozygous carrier.

Table 2 summarizes the genetic testing results and main clinical features of the proband’s relatives.

**Figure 2:**
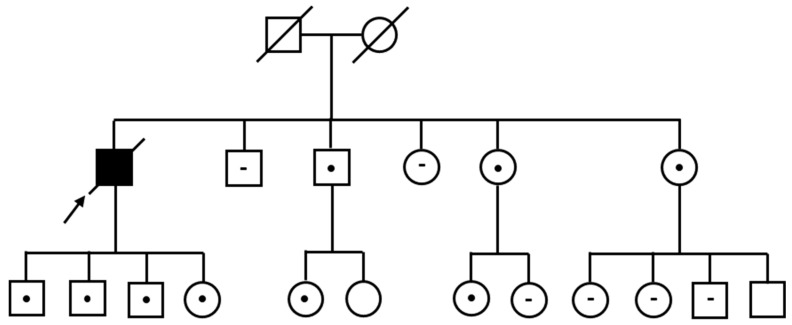
Pedigree of the Calabrian family carrying the *TTR* p.Val142Ile variant. The proband (indicated by the arrow) was found to be homozygous for the c.424G>A (p.Val142Ile) mutation. Individuals carrying the heterozygous variant are marked with a black dot (●), while wild-type individuals are indicated with a minus sign (−). Squares represent males and circles represent females; slashed symbols indicate deceased individuals. Black-filled symbols identify individuals with clinically documented cardiac involvement.

All genetic testing results are summarized in Figure 2.

## 4. Discussion

ATTRv is a systemic disorder caused by pathogenic mutations in the *TTR* gene, leading to destabilization of the transthyretin tetramer, misfolding of monomers, and aggregation into insoluble amyloid fibrils. Once a pathogenic variant in the *TTR* gene has been identified, treatment is primarily symptomatic and focuses on improving the patient’s quality of life. For example, fluid retention is common and should be managed with diuretic therapy. A cardiology consultation is strongly recommended to optimize heart failure management and to evaluate for arrhythmias or conduction abnormalities, which may be present even in the early stages of the disease [19]. Recently, results from the “ATTR-ACT” trial have been published, representing the first randomized study evaluating Tafamidis (commercially known as Vyndaqel) for the treatment of both wild-type and hereditary forms of transthyretin cardiac amyloidosis. Tafamidis is an orally administered small molecule that binds to TTR monomers, stabilizing the tetramer and preventing its dissociation—a key step in the formation of amyloid fibrils [21]. The use of Tafamidis has proven effective in controlling both neurological and cardiac manifestations of the disease, with reductions in all-cause mortality, cardiovascular-related hospitalizations, and a slower decline in functional capacity and quality of life compared to placebo.

The disease exhibits marked genetic and clinical heterogeneity, with mutation-specific phenotypes and distinct geographic distributions [22].

Among the more than 150 known *TTR* variants, p.Val142Ile is one of the most clinically relevant mutations, particularly due to its association with late-onset cardiac amyloidosis. This variant is found in 3–3.5% of African Americans, but its frequency in Caucasians is much lower, around 0.44%, and it is virtually absent in Hispanic populations [23]. Despite its autosomal dominant inheritance, p.Val142Ile is characterized by incomplete penetrance: long-term studies have shown that fewer than 7% of carriers develop overt cardiac amyloidosis over a 20-year period [23].

Although classically linked to individuals of West African ancestry, p.Val142Ile has been increasingly reported in Europe, including Italy, likely due to historical migration and genetic admixture. In recent years, the variant has been identified in Southern Italian patients, particularly from Sicily, where a cohort of 12 Caucasian subjects from seven unrelated families was described [24]. Interestingly, while p.Val142Ile is typically associated with a cardiac-dominant phenotype, several Sicilian patients also presented with peripheral and autonomic neuropathy, and carpal tunnel syndrome (CTS) emerged as a frequent early manifestation. These findings underscore the phenotypic variability of the variant and challenge the previous assumption that it exclusively causes cardiomyopathy.

Calabria, another Southern Italy region, has historically been associated with a different endemic *TTR* variant—p.Phe84Leu (classically p.Phe64Leu)—which has been repeatedly observed in both symptomatic and asymptomatic individuals of Calabrian origin [25].

Our study describes the first documented case of a large multigenerational Calabrian family carrying the p.Val142Ile variant, thereby expanding the known mutational spectrum of ATTRv in Southern Italy.

The proband, homozygous for the p.Val142Ile variant, presented with a cardiac-restricted phenotype characterized by hypertrophic cardiomyopathy and conduction abnormalities. Homozygosity for this mutation is exceedingly rare and has only been reported in isolated cases. In a previously described Sicilian patient, homozygosity was associated with an earlier onset and more severe cardiac involvement [26]. However, in our case, homozygosity for the p.Val142Ile variant did not correspond to a more severe clinical phenotype compared to typical heterozygous carriers, indicating considerable variability in disease expression even in homozygosity. A previously published case of homozygous p.Val122Ile (equivalent to p.Val142Ile) in an African American patient presenting with late-onset cardiomyopathy has been reported, supporting the notion that homozygosity does not necessarily correspond to earlier or more severe phenotypes [27]. This phenotypic concordance between the homozygous proband and heterozygous carriers suggests the involvement of genetic modifiers influencing onset, penetrance, and disease severity. Previous studies have identified noncoding variants and epigenetic marks in genes such as *RBP4*, *FAM129B*, *SKI*, and *ABCA1* associated with cardiomyopathy risk [28], and ongoing research, including the TTR GWAS project (https://ttrgwas.com/), continues to explore the potential value of broad genomic profiling in families with atypical phenotypic patterns.

Extended family screening led to the identification of numerous heterozygous carriers across three generations, most of whom were asymptomatic at the time of evaluation. This underlines the critical role of cascade genetic testing in detecting pre-symptomatic individuals who could benefit from early monitoring and future therapeutic strategies.

The identification of the p.Val142Ile variant in a native Calabrian family—where the p.Phe84Leu mutation has traditionally been considered the only endemic *TTR* variant—raises important epidemiological considerations. This finding provides the first direct in situ evidence of p.Val142Ile in Calabria, suggesting that current regional mutation prevalence data may underestimate the actual genetic heterogeneity of ATTRv, particularly in areas historically regarded as genetically homogeneous. Furthermore, our results are consistent with emerging evidence from other Italian regions: the Tuscany Rare Disease Registry and a cohort study from Lazio have reported sporadic p.Val142Ile cases in individuals of Southern Italian ancestry [29,30]. These observations collectively point to a broader geographic distribution of the p.Val142Ile variant within Italy than previously recognized. Southern Italy, and Calabria in particular, has a well-documented history of admixture resulting from successive migration waves, including Greek colonization, Roman integration, Arab–Norman rule, and trans-Mediterranean exchanges. Genomic studies confirm that Calabria constitutes a genetically stratified isolate, showing contributions from North African, Middle Eastern, Balkan, and Sub-Saharan ancestries [31]. This complex population history supports the hypothesis that the p.Val142Ile TTR variant found in our case may have been introduced through ancient admixture rather than arising independently. Previous studies have characterized TTR-linked haplotypes in European populations. In Italians from Tuscany, carriers of the p.Val142Ile variant shared a specific haplotype (T-A-T-G at rs3764478/rs72922940/rs1791228/rs1791229), which is uncommon in the general Tuscan population, suggesting a founder effect independent of recent African admixture [32]. In contrast, West African populations show higher frequencies of p.Val142Ile (~2.5%) associated with distinct ancient haplotypes, which dispersed into African American groups via transatlantic migration [33]. Moreover, UK Biobank data have documented the presence of p.Val142Ile in non-African individuals, including those of European, Middle Eastern, South Asian, and Latino ancestry, suggesting the possibility of independent mutational events [34]. Therefore, future haplotype studies focusing on SNPs closely linked to the TTR gene—particularly rs3764478, rs72922940, rs1791228, and rs1791229—may clarify whether the Calabrian p.Val142Ile variant reflects an ancient founder effect or derives from African-linked admixture.

Regarding the p.Phe84Leu variant, which accounts for approximately 21.9% of TTR pathogenic variants identified in Italian patients under follow-up in referral centers [35] and is considered endemic to Southern Italy, no haplotype studies have been conducted to date. Although no evidence currently supports a selective advantage, the possibility remains open, particularly in historical contexts of environmental pressures.

Given the often subtle and late-onset presentation of p.Val142Ile-related ATTRv—typically with isolated cardiac involvement—this mutation remains at high risk of underdiagnosis and is frequently misclassified as hypertensive or hypertrophic cardiomyopathy. These findings underscore the importance of incorporating *TTR* genetic screening into the diagnostic evaluation of unexplained cardiomyopathy, particularly in southern regions like Calabria, where non-canonical mutation profiles may be emerging. The present finding raises a broader consideration in medical genetics. Variants often regarded as population-specific can also appear in distinct ethnic or geographic contexts due to historical admixture. Well-documented examples include the hemoglobin S (HbS) variant, which occurs not only in sub-Saharan Africa but also in parts of Southern Italy and Greece [36]; MEFV mutations associated with Familial Mediterranean Fever in non-Middle Eastern populations [37]; and CFTR variants, whose prevalence and distribution vary significantly across Europe and the Mediterranean basin [38]. These patterns support the implementation of inclusive genetic screening approaches, even outside traditionally recognized endemic regions.

## Figures and Tables

**Table 1:** Summary of the most commonly reported TTR mutations associated with ATTRv across different geographic regions. The distribution reflects regional epidemiological patterns based on clinical and genetic studies [19].

Geographic Region	Most Common TTR Mutation
Portugal, Spain, France	Val50Met
Sweden	Val50Met
Japan	Val50Met
South America (e.g., Brazil)	Val50Met
West Africa	Val142Ile
United States	Val142Ile, Thr80Ala, Val50Met
United Kingdom, Ireland	Thr80Ala
Denmark	Leu131Met
Italy	Ile88Leu
London (UK)	Val142Ile
Caribbean	Val142Ile

**Table 2:** Clinical and genetic findings in the proband’s relatives. The table reports degree of kinship, *TTR* mutation status (WT = wild-type; HZ = heterozygous), and main reported symptoms. Individuals not tested for the familial variant are indicated as “Not tested.”.

Degree of Kinship	Result	Symptoms
Brother 1	WT	apparently good health
Brother 2	HZ	arterial hypertension, hypercholesterolemia, cerebral ischemia, type 2 diabetes mellitus, elevated microalbuminuria levels
Sister 1	WT	hypercholesterolemia
Sister 2	HZ	Hashimoto’s thyroiditis + hypercholesterolemia
Sister 3	HZ	hypotension
Son	HZ	apparently good health
Son	HZ	apparently good health
Son	HZ	apparently good health
Daughter	HZ	extrasystoles
Brother’s 2 daughter 1	HZ	hypercholesterolemia
Brother’s 2 daughter 2	Not tested	-
Sister 2’s son 1	WT	apparently good health
Sister 2’s son 2	Not tested	-
Sister 3’s daughter 1	WT	apparently good health
Sister 3’s daughter 2	WT	apparently good health
Sister 3’s son 1	WT	apparently good health
Sister 3’s son 2	Not tested	-
Cousin	HZ	hypercholesterolemia

## Data Availability

The data that supports the findings of this study are available from the corresponding author (R.P.) upon reasonable request.

## Abbreviations

The following abbreviations are used in this manuscript:

ATTRwt	Wild-type transthyretin amyloidosis
ATTRv	Hereditary transthyretin amyloidosis
T4	Thyroxine
RBP	Retinol-binding protein
PCR	Polymerase chain reaction
AF	Atrial flutter
ECG	Electrocardiogram
ATTR	Cardiac amyloidosis
